# Machine Learning for Thermal Transport Prediction in Nanoporous Materials: Progress, Challenges, and Opportunities

**DOI:** 10.3390/nano15211660

**Published:** 2025-10-31

**Authors:** Amirehsan Ghasemi, Murat Barisik

**Affiliations:** 1 Bredesen Center for Interdisciplinary Research and Graduate Education, University of Tennessee, Knoxville, TN 37996, USA; 2 Mechanical Engineering Department, University of Tennessee at Chattanooga, Chattanooga, TN 37403, USA

**Keywords:** thermal conductivity, thermal transport, nanoporous materials, aerogels, explainable machine learning, interpretable machine learning, deep learning, graph neural networks

## Abstract

Predicting the thermal properties of nanoporous materials is a major challenge that affects their applications in efficient thermal insulation and energy storage. This narrative review discusses the application of machine learning models in nanoporous materials, including covalent organic frameworks, metal–organic frameworks, aerogels, and zeolites. It discusses model advancements, with a focus on predictive accuracy and computational efficiency. This includes models such as convolutional neural networks, graph neural networks, and physics-informed neural networks. This study also addresses the limitations of these data-driven models, including data availability, challenges in maintaining physical consistency, and difficulties in generalizing across various material families. Additionally, it covers emerging approaches such as multimodal and transfer learning, which are explored for their potential to reduce computational costs. Moreover, the benefits of interpretable machine learning methods for understanding underlying physical mechanisms are introduced and highlighted. This review provides comprehensive and practical guidelines for researchers using machine learning approaches in the study and design of nanoporous materials.

## 1. Introduction

The thermal behavior of nanoporous materials is critical for applications in thermal management, energy conversion, and energy storage [1,2]. Depending on their structural properties, these materials exhibit a wide range of behaviors, from ultra-low thermal conductivity in aerogels to relatively high conductivity in crystalline frameworks. This variability makes accurate prediction of their thermal conductivity particularly challenging [1,3,4]. Experimental studies often suffer due to the limited reliability of thermal conductivity measurements, which hinders the establishment of precise structure–property relationships. As a result, computational methods such as molecular dynamics (MD) simulations and first-principles phonon calculations based on density functional theory (DFT) have become essential tools for predicting thermal transport in nanoporous systems [5,6]. However, these methods face limitations in terms of computational cost and scalability, especially when addressing the structural diversity of nanoporous materials.

The emergence of machine learning models has changed the field of predicting material properties [7,8]. While the application of ML models in materials science dates back to the 1990s, the prediction of thermal conductivity using these models gained popularity after 2018, with the emergence of graph neural networks (GNNs) [9]. Recent advances in deep neural networks (DNNs), such as cutting-edge GNNs and physics-informed neural network (PINN) [10] architectures [9,11], along with the growth in the computational power of central processing units (CPUs) and graphics processing units (GPUs), as well materials databases, have created great opportunities for the discovery and design of nanoporous materials with tailored thermal properties.

Using ML models in predicting thermal conductivity represents a significant advancement over traditional methods. Table 1 presents the comparative advantages and limitations of traditional computational methods and ML models. The trade-offs between accuracy, computational efficiency, and generalizability are evident across the different approaches. As Table 1 shows, the actual performance of each of these methods varies based on implementation details. For example, the accuracy of the MD approach depends on the quality of the force field, and it can range from poor for simple potentials to near-DFT accuracy for machine-learned potentials. The DFT-based methods struggle with van der Waals interactions, which are important in many nanoporous materials. Additionally, ML models usually cannot extrapolate beyond their training domain, resulting in varying scalability.

This review paper tries to outline the evolution of machine learning models for predicting thermal conductivity in nanoporous materials, from early models to advanced GNNs and physics-informed ML approaches. It analyzes material-specific insights across different categories of nanoporous materials to reveal structure–property relationships that traditional methods often miss. This review addresses key limitations such as data scarcity, failures in temperature-dependent predictions, and poor transferability in ML models. However, it highlights emerging opportunities in multimodal and transfer learning, and offers practical guidelines for selecting appropriate methods. Additionally, this review discusses the transparency of artificial intelligence (AI) approaches that are designed to explain underlying model mechanisms.

The remainder of this review paper is structured as follows: Section 2 presents the theoretical background and physical principles governing thermal transport in nanoporous materials. Section 3 discusses the development and implementation of machine learning approaches in nanoporous materials. Section 4 addresses the concept of transparency in relation to AI. Section 5 reviews and summarizes the limitations. Section 6 discusses emerging AI approaches and future directions. Finally, Section 7 summarizes the key findings of the discussed data-driven models.

## 2. Theoretical Background

### 2.1. Nanoporous Materials

**Figure 1:**
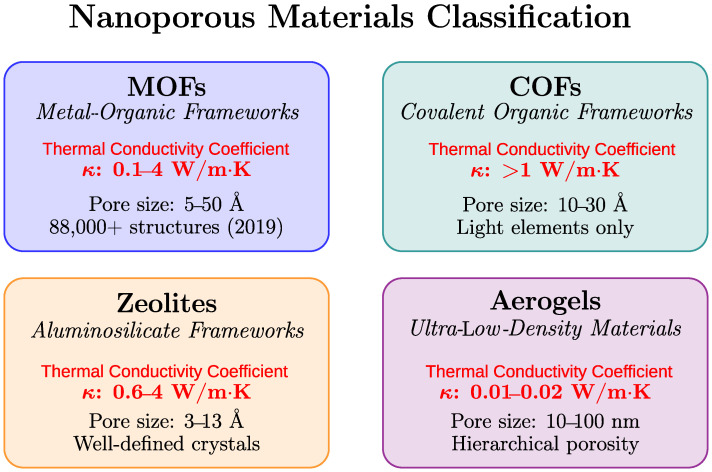
Nanoporous material classifications examined in this study and their corresponding thermal conductivity coefficient (κ) ranges.

Nanoporous materials are characterized by high porosity, a large surface area, and adaptive architectures [1,14,15]. The main categories of these materials include metal–organic frameworks (MOFs), covalent organic frameworks (COFs), zeolites, and aerogels. Each category presents particular challenges in predicting its thermal properties. MOFs consist of metal nodes connected by organic linkers, offering good adaptivity, with nearly 88,000 experimentally determined structures (reported in 2019 [16]; the number increases annually) recorded in the Cambridge Structural Database (CSD). COFs are composed entirely of light elements that are joined by covalent bonds. They provide remarkable stability but are limited by synthetic accessibility [17]. Zeolites possess well-defined crystalline structures; however, their thermal properties are challenging to predict. This is due to the complex interplay between the framework composition and guest molecules [18]. Aerogels are known for their ultra-low density, high porosity, and hierarchical pore structures. They present further complexity because of their unstructured nature and multiple length scales [1]. Figure 1 summarizes the classification and thermal conductivity ranges of the nanoporous materials in this study.

### 2.2. Thermal Transport in Nanoporous Materials

Thermal transport in nanoporous materials occurs across multiple length scales and involves interactions among different heat carriers [1]. The thermal transport regime is governed by the Knudsen number (Kn=λ/H), where λ is the phonon mean free path and *H* is the characteristic pore dimension [19]. For Kn<0.01, transport is diffusive; for Kn>10, it is ballistic; intermediate values represent transitional regimes. In nanoporous frameworks, lattice vibrations, also known as phonons, are the primary heat carriers, although the presence of any guest molecules and electronic conductivity can contribute to additional transport [3]. The effective thermal conductivity is determined by the combined effects of the structural framework, pore architecture, and the presence of any guest species in the nanoscale pores.

Thermal transport in nanoporous materials differs from that in bulk crystalline solids. Nanoscale pores introduce phonon scattering mechanisms that can reduce thermal conductivity compared to their solid counterparts [3,20]. Additional complexity comes from framework–guest molecule interactions, the presence of defects and grain boundaries, and the anisotropic nature of thermal transport in many nanoporous structures [21,22]. Additionally, the transition from diffusive to ballistic phonon transport, which occurs when pore dimensions approach phonon mean free paths, adds more to the complexity [19]. Understanding the abovementioned mechanisms is vital for developing accurate predictive models.

At the microscopic level, nanoscale pores act as additional phonon scattering centers. This substantially reduces the mean free path of heat carriers [23]. This effect becomes more noticeable when pore dimensions approach phonon mean free paths. In bulk silicon at room temperature, phonons with mean free paths exceeding 0.8 μm contribute 37% of thermal conductivity [24]. In nanoporous materials, these long-mean-free-path phonons are truncated by pore boundaries. These dimensional constraints lead to strong phonon confinement effects and alter phonon dispersion relations [25,26]. Additionally, the framework–pore interface introduces additional boundary scattering that depends on surface roughness and the specularity parameter [20]. These microscopic effects can fundamentally challenge traditional continuum models.

Consequently, Fourier’s law of heat conduction, which forms the basis for macroscopic thermal transport descriptions, requires modification when applied to nanoporous materials. At the nanoscale, non-Fourier effects such as ballistic transport and phonon hydrodynamics, become important in materials with highly ordered pore structures [27]. The Boltzmann transport equation is able to provide a more rigorous framework for describing phonon transport in these settings since it can account for the wave nature of phonons and different scattering mechanisms [19]. This theoretical framework is crucial for understanding when classical approximations are insufficient and when more sophisticated treatments are necessary.

Recent studies have examined thermal transport in nanoporous materials with periodic pore structures to understand the role of phonon coherence effects [28,29]. When pore spacing is comparable to phonon wavelengths, periodic structures can create phononic band gaps that restrict thermal transport at specific frequencies. However, experimental measurements at room temperature have shown that classical boundary scattering predominates over coherent effects for structures with feature sizes larger than 100 nm [29]. Additionally, direct comparisons between periodic and aperiodic nanostructures have demonstrated identical thermal conductivities. This indicates that phonon coherence plays a negligible role in a regime of this size. The reduction in thermal conductivity compared to bulk solids results from diffuse phonon scattering at pore boundaries. These findings suggest that particle-based models accurately describe thermal transport in nanoporous materials without requiring wave interference considerations at temperatures above 14 K [29].

### 2.3. Structure–Property Relationships

The relationship between structural features and thermal conductivity in nanoporous materials is complex and often non-intuitive. While intuition suggests that more pores mean lower thermal conductivity, because there is less solid material to carry heat, recent research has proven otherwise. It has shown the importance of features such as pore size distribution, pore connectivity, and framework composition [1,30,31]. For instance, silica aerogel composites have very low thermal conductivities (0.01–0.02 W/m·K) because their nanoporous structure inhibits gas atom movement and limits heat flow through the solid skeleton [1]. On the other hand, some single-crystal covalent organic frameworks (COFs) have demonstrated the ability to conduct heat effectively (>1 W/m·K) even with a nanoporous structure. This is because their strong, connected COF ribbons form a continuous crystal and facilitate efficient heat transfer even when the porosity percentage is high [30]. These contrasting behaviors illustrate how the structural framework can overshadow simple porosity considerations.

Framework topology is an important factor in determining thermal transport properties [32]. Materials with highly connected frameworks, indicated by high coordination numbers at the nodes, typically have higher thermal conductivities. Regarding MOFs, a recent computational screening of over 10,000 hypothetical structures revealed that four-connected metal nodes consistently yielded the highest predicted thermal conductivities, with approximately 65% of high-performance MOFs featuring four-connected nodes. Simulations suggest that these structures can achieve thermal conductivities of up to 2–4 (W/m·K) experimentally, although a small subset shows computational predictions above 10 (W/m·K). In contrast, structures with 12-connected nodes typically exhibit values below 2 (W/m·K) [4,33]. Additionally, pore dimensionality can further affect thermal transport. One-dimensional channels often exhibit highly anisotropic properties [3].

The chemical composition of the framework adds complexity to structure–property relationships. Larger mass differences between atomic species increase phonon scattering and typically lower thermal conductivity [4,34]. Stronger covalent bonds enable more efficient thermal transport. In MOFs and COFs, the organic linkers act as thermal resistances. Their length, stiffness, and functional groups all contribute to this effect [21,35]. In zeolites, non-equilibrium MD simulations have revealed thermal conductivities ranging from 0.6 to nearly 4 (W/m·K) at 350 K, with values varying between different framework types and crystallographic directions [19,36]. Guest molecules adsorbed in zeolites typically have a minor influence on thermal conductivity, though water can reduce values by 20–40% through phonon scattering [35,36].

Recent findings have identified several surprising structure–property relationships. While disorder typically reduces thermal conductivity, recent studies have identified cases in nanoporous graphene where specific disorder patterns can maintain or slightly enhance thermal conductivity, albeit with precise control of pore arrangements [37]. Another interesting effect arises from the presence of guest molecules within pores, which exhibits a complex behavior known as the “rattle effect”. The rattle effect refers to the rattling vibrations of guest molecules confined within pore spaces which occur when these mobile molecules collide with pore walls and scatter heat-carrying phonons [38]. This phenomenon occurs when fluid–solid collisions scatter phonons. It typically reduces thermal conductivity by up to 30% in narrow pores compared to in vacuum (empty-pore) conditions. However, this effect transitions to a different behavior in larger pores when the vacuum is replaced with a fluid. It leads to an increase in heat transfer [38]. These effects depend on pore size, temperature, and the type of fluid involved. These findings underscore the need for predictive models that capture the full complexity of the structure–property relationships in nanoporous materials.

### 2.4. Challenges in Traditional Computational Approaches

Traditional computational methods for predicting thermal transport in nanoporous materials have several limitations. For example, MD simulations provide atomic-level insights but are computationally expensive. Their complexity ranges from O(N) to O(NlogN) to O(N^2^) depending on the treatment of interatomic interactions and implementation of neighbor-searching algorithms [39]. Big O notation describes computational complexity, where O(N) denotes linear scaling, O(NlogN) denotes log-linear scaling, and O(N^2^) denotes quadratic scaling. Developing reliable interatomic potentials, also known as force fields, for complex nanoporous materials remains an active area of research. Many existing interatomic potentials still fail to capture the interactions that affect thermal transport [40,41]. Force fields struggle with two critical aspects: (1) van der Waals forces, where attractions between atoms and molecules, despite being individually weak, collectively influence how heat moves through the porous framework; (2) host–guest molecule interactions, that is, the complex dynamics between the framework structure and any molecules trapped within the pores. This can scatter phonons and substantially reduce thermal conductivity [35,38,40,41].

First-principles calculations offer higher accuracy but are even more computationally expensive. Usually, their application is limited to small unit cells and simplified models [42]. The calculation of phonon properties from first-principles calculations requires the evaluation of force constants through finite displacement methods or density functional perturbation theory, both of which scale cubically, O(N^3^), with system size [42,43]. For nanoporous materials with large unit cells containing hundreds or thousands of atoms, these calculations become impractical, often requiring weeks of computation on a high-performance computing node.

Beyond computational cost, addressing anharmonic effects presents another major challenge for conventional computational methods. Harmonic approximations perform well for modeling thermal transport at low temperatures; however, accurate prediction of thermal conductivity at higher temperatures requires consideration of phonon–phonon scattering processes [44,45]. Integrating three- and four-phonon scattering increases computational complexity and often requires approximations that can compromise predictive accuracy [44]. These computational bottlenecks, combined with the wide chemical space of nanoporous materials, have motivated the development of alternative approaches that can predict thermal conductivity more efficiently and maybe at a lower cost.

## 3. Evolution of Artificial Intelligence Approaches in Nanoporous Materials

**Figure 2:**
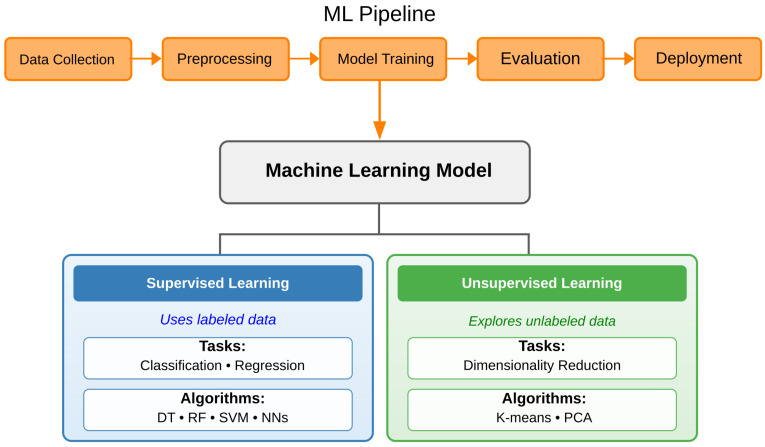
Overview of machine learning categories and typical pipeline stages.

Machine learning (ML) models use statistical methods to identify patterns in data without explicit programming. The field is divided into supervised and unsupervised learning, as illustrated in Figure 2. Supervised learning uses labeled data to train algorithms for classification and regression tasks. Common supervised algorithms include Decision Trees (DT), Random Forests (RF), support vector machines (SVM), and neural networks (NNs). Unsupervised learning, in contrast, explores unlabeled data to discover hidden patterns and structures in the data [46]. These methods are effective at anomaly detection and dimensionality reduction for data visualization. Most popular unsupervised techniques include K-means clustering for grouping similar data samples and Principal Component Analysis (PCA) for reducing data complexity [47]. A typical ML pipeline consists of five stages: data collection, data preprocessing (cleaning, normalization, feature engineering), model training with cross-validation, model evaluation using metrics like accuracy or R^2^, and, finally, deployment for predictions on new data. This approach ensures reproducible and reliable results across different applications [48].

### 3.1. Early Machine Learning Models

**Figure 3:**
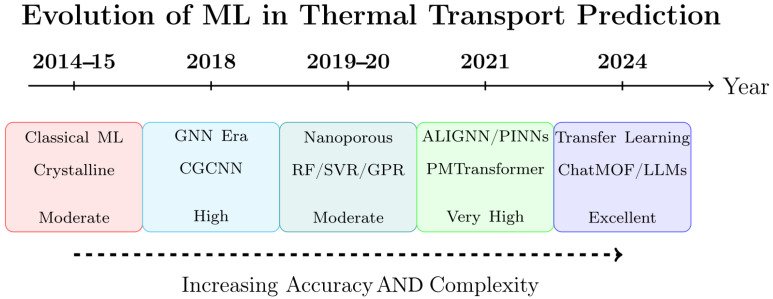
Timeline of the evolution of machine learning (ML) approaches for thermal transport prediction.

Machine learning applications for thermal conductivity prediction in crystalline materials first appeared in the mid-2010s [49,50]. Their application to nanoporous materials appeared more recently, around 2019–2020 [51]. Figure 3 illustrates this evolution. It shows the progression from classical machine learning methods to advanced architectures with improving prediction accuracy. The delay in applying ML models to nanoporous materials stemmed from their structural complexity and the limited and mostly unreliable experimental data [52,53]. The initial research primarily established quantitative structure–property relationships using traditional ML algorithms such as Random Forests (RF) [54] and support vector machines (SVM) [55]. These models relied on hand-crafted feature variables derived from crystallographic information such as pore size, surface area, density, and the void fraction [51].

**Figure 4:**
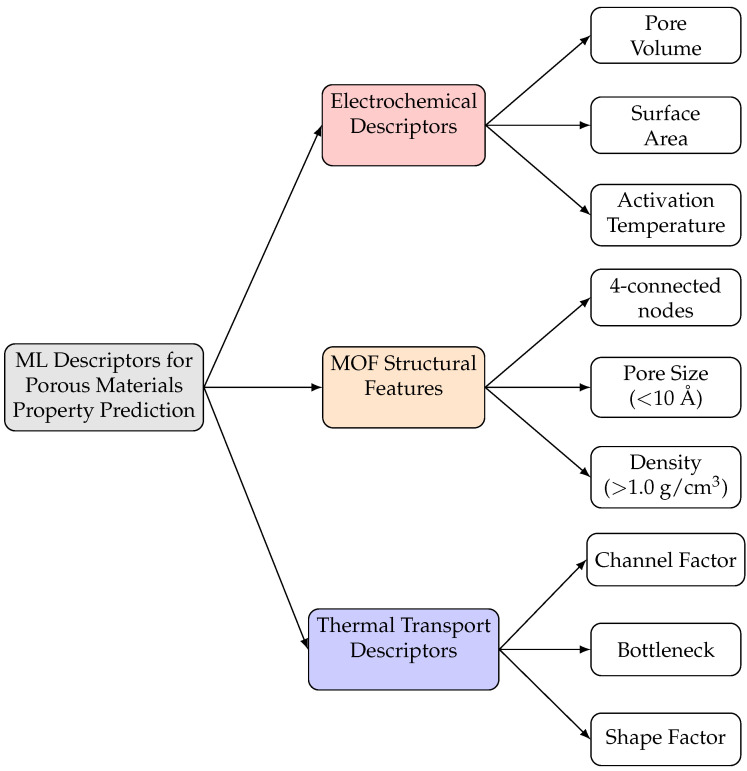
Common descriptors used in ML models for property prediction in porous materials. Wei et al. [51] and Islamov et al. [4] focused on thermal conductivity. Minakshi et al. [57] demonstrated similar structural descriptors applied to electrochemical performance. This highlights the transferability of these features across different applications.

Despite their simplicity, these classical ML models demonstrated good potential for predicting thermal transport properties. For example, Wei et al. [51] used SVR and Gaussian Process Regression (GPR) models to identify five physics-based (features) descriptors. These features included the shape factor, bottleneck, channel factor, perpendicular nonuniformity, and dominant paths, which exhibited strong correlations with thermal conductivity [51]. Random Forest models have also proven effective in related studies due to their ability to capture non-linear relationships and offer built-in feature importance [56]. Studies using these methods achieved reasonable prediction accuracy within their training domain; however, specific performance metrics varied widely depending on the material type and the quality of the data. Similarly, Minakshi et al. [57] used RF and SVR models to predict the specific capacitance of biomass-derived carbon supercapacitors from 166 data samples from the literature. Among the models tested, SVR achieved the highest R^2^ of 0.41398 with a mean absolute error (MAE) of 0.11359, and RF achieved an R^2^ of 0.35689. The results demonstrate that classical ML methods can identify important structure–property relationships in porous materials, with surface area and pore volume showing correlation coefficients of 0.8473. To provide an overview of the descriptors commonly employed in ML-based predictions for porous materials, Figure 4 summarizes key features identified across different application domains.

One major challenge in early ML models was feature engineering, as illustrated by the diverse descriptor types shown in Figure 4. Researchers attempted to use various sets of feature variables, including geometric and chemical features. Choosing the right features required domain knowledge and often repeated testing. Additionally, the fixed-length vectors used in traditional ML algorithms were not suitable for materials with varying sizes and compositions. This necessitated preprocessing that sometimes resulted in the loss of structural information. For instance, encoding the three-dimensional pore network into fixed-length feature vectors resulted in a loss of important connectivity information for thermal pathways. These limitations indicated the need for fundamentally different approaches that could directly process variable-sized crystallographic data while preserving connectivity information [9].

### 3.2. Deep Learning Revolution

Deep learning models in materials research have enabled remarkable capabilities in design, prediction of structure–property relationships, and performance optimization [58,59,60]. DL models have transformed thermal conductivity prediction in nanoporous materials through three major architectures: convolutional neural networks for microstructural analysis, graph neural networks for crystallographic structures, and generative models for inverse design. These approaches overcome the limitations of fixed-length vectors in classical ML while reducing computational costs compared to MD and DFT simulations [61]. DL models have also changed approaches to discovering materials with tailored thermal conductivity for applications ranging from effective thermal insulation to heat dissipation [62].

#### 3.2.1. Convolutional Neural Networks

Convolutional neural networks (CNNs) [63] are recognized as powerful algorithms for analyzing the microstructural characteristics of porous materials through image analysis. CNNs have achieved high accuracy in predicting properties such as porosity, permeability, and tortuosity from two-dimensional (2D) binary images (relative errors < 6%) [64] and the elastic modulus (R^2^ = 0.98) [65], but their direct application to the prediction of thermal conductivity has been limited in the literature.

A study by Du et al. [66] developed a modified AlexNet [67] architecture to predict the effective thermal conductivity of sintered silver. It achieved remarkable accuracy prediction, with an R^2^ value of 0.987. Their model processes 400 × 400 pixel scanning electron microscope (SEM) images through five convolutional layers and predicted thermal conductivities ranging from 25.3 to 422.7 (W/m·K) in just 0.14 s per image. This is reportedly two orders of magnitude faster than finite element simulations [66]. This success was enabled by a robust database of 6,156 realistic microstructures with FE-validated labels (relative error of 5% with experiments). This enabled the CNN model to outperform conventional analytical methods (R^2^ of 0.837) and ML approaches (R^2^ of 0.951).

The advantage of CNNs lies in their translation invariance property, which is achieved through the combination of convolutional and pooling layers. This enables recognition of pores, channels, and connectivity patterns regardless of their spatial location within the microstructure [68,69]. This capability is particularly valuable for porous materials with repeating structural features. CNNs can analyze entire samples using a single set of convolutional filters rather than requiring position-specific feature extractors. Therefore, they can provide computational robustness and efficiency in the case of variations in sample positioning.

For three-dimensional (3D) analysis, CNNs process volumetric data from techniques such as micro-CT scanning [69] and capture the complete pore network structure. Santos et al. [70] developed PoreFlow-Net, a 3D CNN architecture that predicts velocity fields in porous media with speedups of several orders of magnitude compared to numerical simulations. Kamrava et al. [69] demonstrated that 3D convolutions enable accurate predictions of permeability for realistic pore geometries. In a comprehensive study, a 3D CNN was used to analyze 90,000 artificially generated microstructures and establish robust microstructure–property relationships for diffusivity and permeability [71].

Despite these advantages, CNN-based approaches have limitations. The application of CNNs to disordered nanoporous materials remains challenging due to ill-posed problems, non-linear correlations, and a lack of interpretable features [72]. While CNNs excel at image segmentation and structural characterization [72], their success in predicting thermal conductivity has been demonstrated for materials with semi-ordered structures, such as sintered metals [66]. The Du et al. model [66] also proved cross-material transferability. It successfully predicted the thermal conductivity of sintered Cu (R^2^ = 0.975) by using a model trained on Ag data (Ag-trained model). This suggests the potential of the model for broader applications within similar material classes.

However, the training requirements for DL models vary significantly, from 1000 samples for specific properties [65] to 90,000 samples for comprehensive transport property prediction [71]. Furthermore, prediction accuracy can degrade when models encounter microstructures outside their training distribution, and errors increase for irregular pore geometries [65].

#### 3.2.2. Graph Neural Networks

Graph neural networks (GNNs) [9,12] have advanced the analysis of nanoporous materials by capturing the molecular structure and chemical bonding patterns. Unlike traditional descriptor-based approaches, GNNs can directly process three-dimensional atomic arrangements and chemical connectivity. It can handle the variable sizes and periodic boundary conditions inherent to crystal structures. By representing materials as graphs with atoms as nodes and chemical bonds as edges (also known as links), GNNs learn hierarchical features through message-passing (embedding) frameworks throughout the molecular graph. Therefore, they can capture local and global structural features relevant to thermal transport [73,74,75].

Subsequent developments in GNN architectures further enhanced their applications in predicting thermal conductivity. The inclusion of edges with features that represent bond types, lengths, and angles provides additional chemical context and improves prediction accuracy [76]. Also, advancements in pooling strategies have enabled GNNs to learn multi-scale representations that capture both local atomic and global structural features [77,78].

The crystalline nature of MOFs and COFs is described by well-defined atomic positions and bonding patterns. It makes them ideal candidates for GNN architectures. This contrasts with the ill-defined connectivity observed in disordered materials. The crystal graph convolutional neural network (CGCNN) [9] architecture is specifically designed for crystalline materials. It has demonstrated remarkable success in predicting the thermal conductivity of lattice structures [9,79]. The CGCNN encodes compositional and structural information through learned atom and bond features, which are aggregated across multiple convolutional layers to produce material-level predictions [11]. The ability of the CGCNN to achieve high accuracy with relatively small training sets has made it attractive for studying materials where experimental data are often limited.

The enhanced crystal graph convolutional neural network (ECGNN) represents a significant architecture advancement. It incorporates attention mechanisms (multi-head attention) and geometric features to achieve remarkable performance in gas adsorption prediction. It has been demonstrated that the method predicts CO_2_ adsorption properties in MOFs with accuracies comparable to those of Grand Canonical Monte Carlo (GCMC) simulations, which can take hours to days, thereby reducing computational time by almost five orders of magnitude [73].

**Figure 5:**
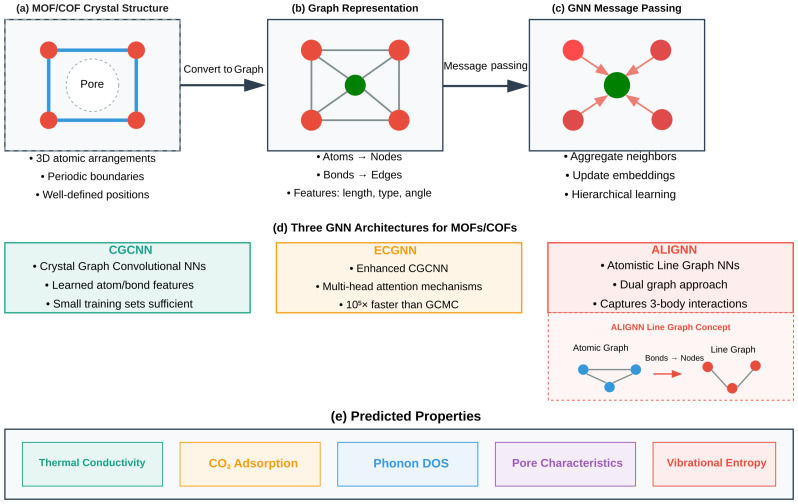
(**a**) MOF/COF crystal structure with periodic boundaries. (**b**) Graph representation with atoms as nodes and bonds as edges. (**c**) Message-passing mechanism for hierarchical feature learning. (**d**) Three GNN architectures: the CGCNN, ECGNN, and ALIGNN with its dual-graph approach. (**e**) Predicted thermal and adsorption properties.

The atomistic line graph neural network (ALIGNN) integrates atomic graphs and their corresponding line graph representations [11]. This dual approach captures angular information and three-body interactions, which are essential for accurate property prediction. The ALIGNN has been used to predict the CO_2_ adsorption of MOFs. It has also achieved high-accuracy prediction for properties such as adsorption isotherms, surface area, and pore characteristics [80]. The model has also shown promise in predicting the phonon density of states (DOS) and derived thermodynamic characteristics such as heat capacity and vibrational entropy [81]. Figure 5 illustrates the three main GNN architectures (the CGCNN, ECGNN, and ALIGNN) that have advanced the analysis of nanoporous materials.

#### 3.2.3. Generative Models

The application of generative models to nanoporous materials design represents a significant advancement from classical trial-and-error approaches to systematic, data-driven materials discovery [62]. Generative adversarial networks (GANs) [82] and variational autoencoders (VAEs) [83] have emerged as robust architectures for inverse design applications, where desired properties guide the generation of corresponding material structures [84].

A study by Du et al. [85] employed conditional GANs (CGANs) to generate microstructures of sintered silver with the desired effective thermal conductivity. Their model achieved a prediction accuracy of R^2^ = 0.985 between the target and CNN-predicted thermal conductivities when conditioned on both thermal conductivity and porosity. The CGAN could generate multiple microstructure candidates within seconds (0.2 s per image). Each image displayed a realistic pore morphology that achieved the target thermal properties. This inverse design approach, which begins with a desired thermal conductivity and generates corresponding structures, demonstrates the potential of generative models for developing materials with tailored thermal properties.

Generative models have been effective in generating realistic microstructures for various applications. Recent implementations of supramolecular VAEs have demonstrated the ability to generate MOFs with superior gas separation performance that outcompete some of the best-performing materials ever reported [84]. In terms of mechanical properties, GAN-CNN frameworks have been used to create optimized nanoporous silicon nitride membranes, demonstrating 32% higher strength compared to conventional cubic pore patterns. This was achieved through reduced stress concentration factors around pore edges [60].

Generative models ensure that generated structures retain physical realism while exploring novel design spaces that may not exist in traditional databases [62,84]. The success of Du et al.’s model [85] suggests that generative models could be extended to other thermal properties and more complex disordered structures, though challenges remain in ensuring that generated structures are both thermally optimal and synthetically accessible. Future work might combine generative models with physics-informed constraints to ensure that proposed structures respect fundamental thermal transport mechanisms while maintaining manufacturability.

#### 3.2.4. Physics-Informed Neural Networks

Physics-informed neural networks (PINNs) [10] encode fundamental physical laws and constraints directly into the learning (training) process. The idea of integrating physical equations into neural networks (NNs) dates back to 1998, when it was used to solve differential equations [86]. The modern PINN framework was formalized in 2019. By encoding governing equations (ODEs, PDEs) into the neural network’s loss (cost) function, PINNs ensure predictions respect conservation laws and thermodynamic principles while addressing the limitations of traditional NNs in extrapolation and physical consistency [87,88].

Recent work has demonstrated the potential of PINNs for predicting thermal conductivity in porous materials. Liu et al. [89] applied PINNs to solve inverse problems in polyurethane–PCM foam composites. They accurately predicted spatially varying thermal conductivity fields from temperature measurements, with an R^2^ value exceeding 0.99, even with noisy sensor data. This hierarchical multi-scale approach represents one of the few successful applications of PINNs to thermal transport in porous materials, although it operates at the continuum scale rather than the phonon transport scale.

**Figure 6:**
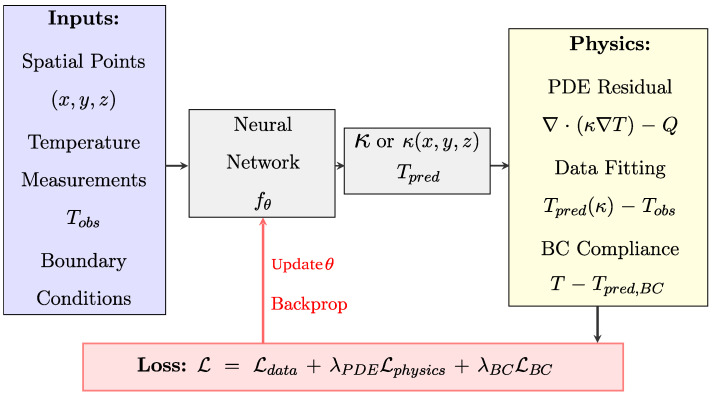
Proposed physics-informed neural network (PINN) architecture for inverse thermal problems in nanoporous materials. The network processes spatial coordinates (x,y,z) and sparse temperature measurements Tobs to infer the unknown thermal conductivity κ(x,y,z) or $T_{pred}$. The loss function combines the data fitting error, physics residuals from the heat diffusion equation, and boundary condition constraints to ensure physically consistent predictions.

We propose a PINN architecture in Figure 6 that could be adapted for inverse thermal conductivity identification in nanoporous materials. It builds upon the established PINN framework for inverse problems [10]. The network processes spatial coordinates (x,y,z) and sparse temperature measurements $T_{obs}$ to infer the unknown thermal conductivity, either as an effective value $\kappa_{eff}$ or a spatially varying field κ(x,y,z). The multi-objective loss function $L=L_{data}+\lambda_{PDE}L_{physics}+\lambda_{BC}L_{BC}$ [10] enforces consistency between predictions and observations. $L_{data}$ minimizes the discrepancy between predicted and measured temperatures. Additionally, $L_{physics}$ ensures the inferred conductivity satisfies the heat equation ∇·(κ∇T)−Q=0, and $L_{BC}$ enforces boundary conditions. We propose that this inverse approach would be particularly valuable for characterizing nanoporous materials where direct measurement of effective thermal properties is challenging due to size effects and measurement limitations.

PINNs have also shown success in modeling flow and transport phenomena in porous media by incorporating Darcy’s law and advection–dispersion equations [90,91,92,93]. These applications demonstrate PINNs’ ability to bridge data-driven learning and mechanistic understanding; however, their application to nanoscale thermal transport in disordered nanoporous materials remains largely unexplored. The challenge lies in encoding the phonon transport mechanisms and size effects that dominate at nanoscales, which differ fundamentally from continuum heat diffusion.

While PINNs’ computational costs remain higher than those of purely data-driven approaches [93], this gap represents an opportunity for future research, as physics-informed approaches could potentially address the extrapolation challenges faced by conventional ML methods in predicting the thermal conductivity of disordered nanoporous materials.

## 4. Transparency in Artificial Intelligence

The arrival of predictive AI models has contributed to solving engineering problems. Based on the problem, AI models can be applied to data to make predictions. However, complex AI models are considered black boxes [94] and lack transparency. Furthermore, real-world problems are complex and demand complex models. Many DNN architectures, due to neural networks’ universal approximation capabilities [95,96], can capture complex relationships in data. Therefore, they can be a good remedy for these complex problems. However, as mentioned, complex models need transparency.

AI models, such as ensemble learning approaches, SVM, and DL models, are categorized as “black-box” models. In contrast, simpler models, such as K-nearest neighbors (KNN) and linear regression models, are intrinsically explainable and known as “white-box” models. White-box models are less accurate than black-box models and usually do not perform well for complex problems. Therefore, the trade-off between accuracy and transparency should be considered when choosing an AI model.

### 4.1. Explainable AI

**Figure 7:**
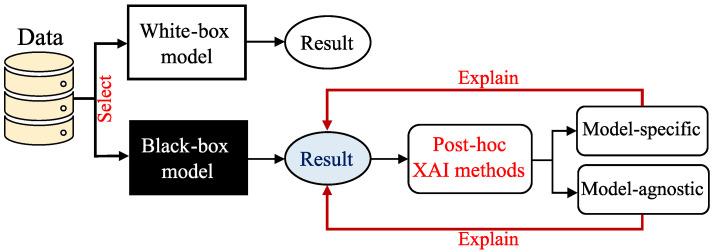
AI models and XAI methods.

eXplainable Artificial Intelligence (XAI) [97] is a collection of methods for understanding the predictions of AI models. XAI employs a post hoc approach and incorporates several explanatory methods, thereby making the results of the black-box model explainable. The complexity of an AI model can be restricted by choosing white-box models to provide explanations without applying XAI methods [94]. However, selecting a black-box model needs explanations. As Figure 7 depicts, the post hoc approach explains the AI model results after the training and inference processes [98,99]. The post hoc approach includes several XAI methods, known as “model-specific” methods, that have been developed to explain a specific AI model architecture. For instance, class activation mapping (CAM) [100] and gradient-weighted class activation mapping (Grad-CAM) [101] explain CNNs by creating a heatmap where the hot part corresponds to a particular class. The other XAI methods in the post hoc approach, known as “model-agnostic” methods, can be implemented for any black-box estimator.

Generally, a post hoc method is preferred over an intrinsic model since it applies to a black-box model’s prediction and, commonly, black-box models (complex models) perform better. It should be noted that post hoc methods can also be applied to a white-box model’s result and provide more transparency [94]. Additionally, an analysis of the literature on XAI reveals that most existing XAI methods applicable to tabular data, the most common data type [102], are model-agnostic [103]. Post hoc models such as Shapley Additive exPlanations (SHAP) [104], Local Interpretable Model-Agnostic Explanations (LIME) [105], and Anchors [106] use feature ablation and permutation techniques to estimate feature importance. They compute how the model prediction error changes when an attribute (feature) is taken out, or its values are shuffled [103].

Other model-agnostic techniques, such as unconditional counterfactual explanations [107], are considered counterfactual explanations. They look for the smallest changes in features that can shift a prediction to a different preferred result [103]. Moreover, some model-agnostic methods, such as Partial Dependence Plots (PDP) [108], highlight the relationship between the target and input and provide a visual explanation.

### 4.2. Interpretable AI

**Figure 8:**
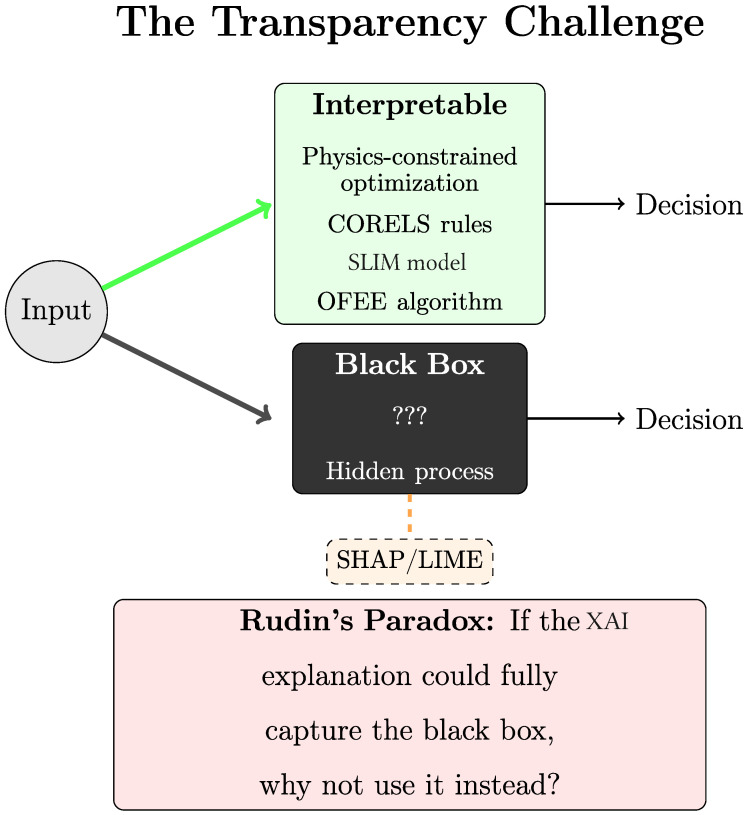
The fundamental challenge of interpretability. Physics-constrained optimization, CORELS, and OFEE provide transparent models. Black-box models require post hoc explanations.

A fundamental distinction exists in AI research between interpretable and explainable AI [98]. Rudin (2019) [109] characterizes interpretable AI as models that are inherently transparent. These models enable users to understand the decision-making mechanism of AI models. In contrast, explainable AI employs post hoc explanation methods that can be applied to AI models after predictions have been made. These XAI methods attempt to analyze the decision logic of complex models by examining the inputs and outputs that approximate the AI’s behavior. As Rudin argues (Figure 8), using XAI for the explanation of black-box models faces an inherent paradox: Post hoc XAI methods must offer explanations that are simpler than the AI model, so they can be useful for the user. However, this simplification means they are insufficient to fully capture the AI model’s behavior. If they could fully capture the AI model’s behavior, the complex AI model itself would be unnecessary [109].

The assumption that predictive accuracy decreases with model interpretability has been challenged by recent studies. Rudin demonstrated that this trade-off does not consistently occur, particularly in structured datasets with semantically meaningful features [109]. Evaluations have shown that interpretable models such as certifiably optimal rule lists (CORELS) [110], which implement if–then rule structures, and sparse scoring systems using integer coefficients, known as Supersparse Linear Integer Models (SLIMs) [111], achieve comparable performance metrics to “black-box” models while maintaining full transparency [109].

Lazebnik et al. [112] extended this concept with the OFEE algorithm. It is designed to balance transparency and performance as an optimization problem rather than a fixed trade-off. OFEE quantifies explainability using metrics such as ensemble size and stability rather than treating interpretability as a binary concept. The algorithm achieved an 8% improvement in size-based explainability and a 7% improvement in stability-based metrics compared to other feature selection algorithms. The OFEE algorithm is parameter-free and self-tunes its hyperparameters, which allows for the adaptive balancing of accuracy and interpretability across various datasets.

Deep symbolic regression has also been identified as an interpretable AI [113]. It reveals the underlying mathematical equations based on the most correlated features targeted [113]. Although these equations are symbolically explicit and mathematically accurate, they may lack the intuitive physical meaning necessary for scientific practice [114]. As a result, such expressions, while formally transparent, can remain opaque if they do not align with domain knowledge.

Surrogate models provide a strategy for achieving interpretability by constructing simplified approximations of complex black-box AI models. However, a surrogate cannot simultaneously be simpler than the original and perfectly replicate its behavior. This limitation introduces uncertainty regarding the reliability of either model [94].

A promising approach involves physics-constrained optimization, which integrates domain knowledge into the optimization process. Theory-guided data science (Karpatne et al. [115]) explains how incorporating physical laws, either as hard constraints that restrict solutions to physically feasible spaces or as regularization terms that guide solutions toward physical consistency, can produce interpretable models. These models are interpretable because their structure inherently reflects the governing physical laws. As Rudin and Karpatne et al. emphasize, genuine interpretability must be domain-specific, with models that respect the domain’s structural knowledge.

### 4.3. Ethical and Societal Considerations

Using AI in materials research faces several challenges. When advanced AI resources are primarily available to well-funded institutions, existing gaps in the field can widen. The accessibility of AI tools and training datasets is a crucial factor for creating a diverse and inclusive research community. There are also environmental concerns that must be taken into consideration: Training large AI models requires a significant amount of energy. While AI can help identify materials for energy-efficient technologies, the carbon emissions associated with these processes need to be considered [116]. Using more efficient training methods and renewable energy for computing is an important step toward making data-driven materials research more sustainable.

### 4.4. Applications of Explainable/Interpretable AI in Nanoporous Materials

The application of explainable AI in predicting the thermal conductivity of nanoporous materials remains largely confined to the post hoc implementation of existing XAI methods rather than the development of domain-specific interpretable approaches. The literature has demonstrated the use of the SHAP algorithm on models like XGBoost to analyze feature importance after model training [117]. Even when molecular descriptors are employed for materials like zeolites, researchers typically extend existing methods, such as SHAP, through assignment techniques rather than creating fundamentally new interpretability frameworks [118]. Studies on polymeric graphene-enhanced composites similarly rely on SHAP model integration to determine the local/global influence of features on estimations [119]. Some works incorporate physical descriptors that are inherently more interpretable than traditional graph descriptors, or employ symbolic regression to construct mathematical formulas [120]. Symbolic regression approaches may reveal new scaling laws for nanoporous materials [121]. These still represent applications of established XAI techniques rather than novel methodologies tailored to the unique physics of thermal transport in nanoporous materials. This limitation suggests that the field has yet to develop specialized interpretability methods that provide deeper insights into the complex phonon scattering mechanisms, size effects, and structure–property relationships specific to thermal conductivity in nanoporous systems.

## 5. Limitations

Despite remarkable advances in machine learning applications for predicting thermal transport in nanoporous materials, several fundamental limitations still constrain these approaches.

The most critical challenges relate to data availability and quality. Experimental measurements of thermal conductivity in nanoporous materials are challenging, time-consuming, and often inaccurate [53]. This results in sparse datasets with significant uncertainties [52,53] which typically contain fewer than 1000 samples, limiting the model’s generalizability [122]. The heterogeneity of available data adds more complexity to this challenge. It is reported that these disparities may be attributed to variations in measurement conditions, sample preparation, and material morphology. For instance, the thermal conductivity measurements of MOFs can differ by orders of magnitude between powders, pellets, and single crystals due to grain boundary effects and variations in packing density [4,35]. High-throughput computational approaches have been used in an attempt to generate larger datasets for thousands of structures [4,123]; however, systematic comparisons revealed significant discrepancies between the computational predictions and experimental values [52]. Therefore, this limits their reliability as training data samples for ML approaches.

The role of porosity in ML prediction accuracy remains complex and multifaceted. While porosity-related features such as pore volume and surface area often appear as important descriptors in ML models [51,57], their direct impact on prediction accuracy varies significantly. Wei et al. [51] identified geometric descriptors, including shape factor and channel factor, as influential for thermal conductivity prediction in porous media. The relationship between porosity and thermal conductivity in nanoporous materials is non-linear, as thermal transport transitions from diffusive to ballistic regimes depending on the ratio of the phonon mean free path to the pore dimensions [3,19]. The challenge lies in capturing the multi-scale nature of porosity and its effects on thermal transport [1]. Future work should systematically investigate how varying porosity levels and pore size distributions specifically impact ML model accuracy.

Current ML models face extrapolation challenges. Even state-of-the-art architectures show substantial performance degradation when predicting the properties of materials outside their training domains [124,125]. Models trained on specific topologies require complete retraining for different nanoporous frameworks, and predictions for materials with extreme property values often fail entirely. Sometimes, this results in errors increasing several-fold [121,126]. Temperature-dependent thermal conductivity remains particularly challenging. While some approaches can predict thermal conductivity at specific temperatures, capturing the full temperature dependence requires either training separate models for each temperature or developing sophisticated architectures that explicitly account for temperature as a variable [127]. The inclusion of the anharmonic effects and phonon–phonon scattering processes that govern temperature dependence adds considerable complexity to this process [128].

The computational demands of advanced approaches, particularly deep learning models, present an additional challenge. For example, training graph neural networks or physics-informed models may require days or weeks on high-performance computing nodes and is limited to well-funded institutions. While inference is typically fast once models are trained, the need for computational resources for retraining when new data become available creates ongoing computational bottlenecks.

The experimental validation of AI models presents a significant set of challenges. Many predicted structures, particularly those from inverse design approaches, prove difficult or impossible to synthesize with current techniques [16]. Even successful syntheses often yield materials that deviate significantly from idealized computational models due to defects, grain boundaries, and other imperfections, which significantly affect thermal conductivity [35].

The sensitivity of thermal conductivity to measurement conditions introduces additional challenges for validation. Factors such as sample density, grain size, moisture content, and measurement atmosphere can all influence measured values [21,33]. AI models trained on computational data or measurements under idealized conditions often fail to accurately predict thermal conductivity under realistic operating conditions. This limits their practical utility for materials design and engineering applications.

## 6. Emerging Opportunities and Future Directions

### 6.1. Transfer Learning and Domain Adaptation

Transfer learning (TL) is a powerful strategy for addressing data scarcity in DL approaches. The implementation of TL follows a two-step process: Initially, a base model is trained on a large dataset of materials with known properties. It learns general features and patterns that govern the underlying physics. Then, this pretrained model is fine-tuned on a smaller dataset which is distinct to the target material class to learn and predict characteristics of the new material domain. This approach has performed significantly in nanoporous materials. Park et al. [129] showed that their porous material transformer (PMTransformer) model, which was pretrained on 1.9 million hypothetical porous materials, could predict properties of COFs, including gas uptake, heat of adsorption, and band gaps, despite limited training samples. Studies have also demonstrated quantitative improvements specifically for the prediction of thermal conductivity. Elmorsy et al. [130] achieved a 70% accuracy improvement with a 2.8-fold faster training time in digital porous rocks. Pai et al. [131] improved R^2^ values from 0.83 to 0.93 in thermal conductivity prediction in nanofluids. Chang et al. [132] introduced the “mixture-of-experts” framework, which outperformed traditional TL in 14 of 19 material property tasks.

Beyond TL, emerging cross-property frameworks offer new opportunities for predicting thermal conductivity. Gupta et al. [133] demonstrated that models trained on formation energy can successfully predict diverse properties such as elastic moduli and dielectric constants. Their model outperformed scratch models in approximately 69% of cases, despite using only elemental fractions as input. While thermal conductivity was not directly tested, the fundamental physics encoded in formation energy models are highly relevant to thermal transport. This cross-property approach could be especially valuable for nanoporous materials where thermal conductivity data are limited but related properties have been studied.

Recent architectural advances hold promise for efficient transport properties in TL. For example, transformer-based models with self-attention mechanisms have demonstrated exceptional data efficiency [134]. Meng et al. achieved an R^2^ of 0.9563 using only 300 training samples for permeability prediction [134]. Future work should explore optimal source property selection and domain adaptation strategies specifically tailored for thermal transport in nanoporous materials.

### 6.2. Multimodal Learning

Multimodal learning integrating structural data with synthesis conditions and characterization results represents a promising frontier. Early work combining synthesis parameters with structural descriptors showed improved prediction accuracy [125]. Recent developments in multimodal learning have enabled the integration of diverse data sources for enhanced property prediction. The porous material transformer (PMTransformer) has demonstrated this, achieving state-of-the-art performance using multimodal transformer models pretrained on 1.9 million porous materials [129]. These approaches combine imaging data from different sources and with different resolutions to create detailed three-dimensional characterizations of porous materials. Multimodal learning techniques have proven particularly effective in reducing data requirements and improving prediction efficiency, demonstrating strong performance even with limited training datasets [129,135]. The development of large language model (LLM)-based approaches represents an emerging frontier in nanoporous materials research. ChatMOF, an AI system leveraging Generative Pretrained Transformer 4 (GPT-4) [136], has demonstrated accuracy rates of 96.9% for searching, 95.7% for predicting, and 87.5% for generating tasks, successfully creating materials with user-desired properties from natural language inputs. This approach significantly reduces the barriers to computational materials design by eliminating the need for specialized programming knowledge [137].

### 6.3. Other Approaches

Active learning strategies integrated with autonomous experimentation systems could accelerate discovery by orders of magnitude. Recent demonstrations have shown that autonomous systems can discover materials with targeted thermal properties significantly faster than traditional approaches [138]. Federated learning frameworks could address data scarcity while preserving intellectual property, enabling collaboration between research groups unable to share raw data directly [139].

Quantum-accurate machine learning potentials, while still using classical computing, achieve DFT-level accuracy for molecular dynamics simulations at a fraction of the computational cost [140]. These approaches enable the long-timescale simulations necessary for studying temperature-dependent thermal transport and framework flexibility effects.

## 7. Conclusions

The application of AI in predicting thermal transport and its properties in nanoporous materials has evolved from simple regression models to sophisticated DNN architectures that incorporate physical constraints and domain knowledge. GNNs, particularly the CGCNN and ALIGNN, have achieved great success in capturing structure–property relationships through their ability to process three-dimensional atomic arrangements and chemical connectivity while handling variable-sized input vectors and periodic boundary conditions [9,11]. PINN architectures ensure that predictions respect conservation laws and thermodynamic principles while addressing the limitations of NNs in extrapolation and physical consistency [87,88].

Material-specific findings have emerged from applying ML models to various nanoporous classes. In MOFs, high-throughput screening (HTS) of over 10,000 hypothetical structures revealed that a framework density above 1.0 g/cm^3^ and pore sizes below 10 Å correlate with enhanced thermal conductivity. Ultra-low thermal conductivities below 0.02 (W/m·K) are observed with extremely large pores exceeding 65 Å [4]. For COFs, single-crystal structures demonstrate effective heat conduction (>1 W/m·K) even with high porosity. This is attributed to their strong, connected ribbons forming continuous crystals [30]. In zeolites, non-equilibrium MD simulations reveal thermal conductivities ranging from 0.6 to nearly 4 (W/m·K) at 350 K, with varying values between framework types and crystallographic directions. It is also observed that water adsorption could reduce values by 20–40% through phonon scattering [35,36].

Significant challenges exist in realizing the full potential of AI. Data scarcity for novel materials constrains model development. Many studies rely on datasets containing fewer than 1000 samples [122]. The computational cost of complex models also restricts their accessibility. Additionally, experimental validation remains a bottleneck due to synthesis challenges and unreliable measurement [35,53]. Moreover, temperature-dependent predictions and hierarchical structure representation require further development, as current models struggle to capture anharmonic effects and multi-scale transport phenomena [127].

Looking forward, emerging models show promise. TL approaches, exemplified by the PMTransformer model pretrained on 1.9 million porous materials, have achieved a 70% accuracy improvement with a 2.8-fold faster training time [129,130]. ML interatomic potentials achieve near-DFT accuracy at reduced computational cost, enabling the long simulations necessary for studying temperature-dependent thermal transport [141,142]. Active learning workflows that iteratively select structures based on model uncertainty have proven effective for efficiently exploring material space [143].

The development of interpretable AI methods for materials science will be significant for building trust in model predictions. Deep symbolic regression approaches and interpretable models such as CORELS have shown promise for discovering while maintaining full transparency [110,113]. Most importantly, the integration of physics-constrained optimization, which incorporates domain knowledge as hard constraints or regularization terms, can produce models that inherently reflect governing physical laws while achieving high accuracy [115].

Success will require continued collaboration between materials scientists, computer scientists, and experimentalists. The field stands at an inflection point where AI has proven its value but has yet to achieve its full potential. As the challenges of data scarcity, model interpretability, and experimental validation are addressed, AI-driven approaches will enable the design of nanoporous materials with precisely controlled thermal properties. This could revolutionize the field for next-generation efficient thermal management, energy efficiency, and sustainable technologies.

## Figures and Tables

**Table 1:** Comparison of traditional and machine learning-based methods for thermal conductivity prediction.

Method	Training Time	Inference Time	Accuracy	Scalability	Interpretability	Data Requirements
MD Simulations	N/A	High	Low–High	Low	High	Low
DFT+Phonons	N/A	Very High	Medium–High	Very Low	Very High	Low
Classical ML	Low	Very Low	Medium	Medium	Medium	Medium
DNNs	High	Very Low	High	Medium–High	Low	High
GNNs [12,13]	High	Very Low	High	High	Medium	Medium–High
PINNs [10]	Very High	Medium	High	Medium	High	Medium

## Data Availability

Data sharing is not applicable.

## Abbreviations

The following abbreviations are used in this manuscript:

AI	Artificial Intelligence
ALIGNN	Atomistic Line Graph Neural Network
CAM	Class Activation Mapping
CGCNN	Crystal Graph Convolutional Neural Network
CGAN	Conditional GAN
CNNs	Convolutional Neural Networks
COFs	Covalent Organic Frameworks
CSD	Cambridge Structural Database
DFT	Density Functional Theory
DNNs	Deep Neural Networks
ECGNN	Enhanced Crystal Graph Convolutional Neural Network
GANs	Generative Adversarial Networks
GCMC	Grand Canonical Monte Carlo
GNNs	Graph Neural Networks
Grad-CAM	Gradient-Weighted Class Activation Mapping
LIME	Local Interpretable Model-Agnostic Explanations
MD	Molecular Dynamics
ML	Machine Learning
MOFs	Metal–Organic Frameworks
NNs	Neural Networks
PDP	Partial Dependence Plots
PINNs	Physics-Informed Neural Networks
SHAP	Shapley Additive exPlanations
TL	Transfer Learning
VAEs	Variational Autoencoders
XAI	eXplainable Artificial Intelligence
SLIM	Supersparse Linear Integer Model
